# A special jubilee: 100 fake osteosarcoma articles

**DOI:** 10.1016/j.esmoop.2021.100358

**Published:** 2021-12-20

**Authors:** S.S. Bielack, E. Palmerini

**Affiliations:** 1 Cooperative Osteosarcoma Study Group, Pädiatrie 5 (Onkologie, Hämatologie, Immunologie), Zentrum für Kinder-, Jugend- und Frauenmedizin, Stuttgart Cancer Center, Klinikum Stuttgart – Olgahospital, Stuttgart, Germany; 2 Osteoncology, Bone and Soft Tissue Sarcomas and Innovative Therapies Unit, IRCCS Istituto Ortopedico Rizzoli, Bologna, Italy

We are clinical researchers leading some of the largest and most influential study groups on a rare cancer, osteosarcoma. From time to time, we screen the literature to learn what is new for this disease. In recent years, we have had to observe more retraction notices than we were previously used to. Retraction is the action by which a published paper is removed from the journal due to scientific fraud (fabrication, falsification, and plagiarism) or other kinds of misconduct (such as fake peer review). For osteosarcoma, this increase seemed to follow a very much less than arbitrary pattern. We therefore searched pubmed.com for all articles containing the words ‘osteosarcoma’ and ’retraction or retracted’. After excluding unrelated articles and multiple mentions, we analyzed the first 100 eligible articles, until July 2021, by 91 first authors (range: 1-3) from 52 cities in six affected countries. Ninety-six of the retracted publications stemmed from four countries in Asia (83, 10, 2, and 1 papers), three from one country in North America, and one from Europe.

Ninety-eight retracted articles concerned laboratory findings and two clinical–laboratory correlations. Fifty-nine of the articles related to RNA. Fifty-four journals (range: 1-12 retractions) were affected, all had an online subscription option or were open access only. The median time from publication to retraction was 2 years (range: 0-17 years). The median 2021 Journal Impact Factor of 84 papers published in listed journals was 3.849 (range: 1.256-12.531).^1^ There was a clear increase in the number of retracted manuscripts per year: only five appeared before the millennium and 95 thereafter.

Our observation that osteosarcoma retractions are increasing almost exponentially is in line with the situation of health sciences in general.^2^ Retractions which actually occur are probably just the tip of an iceberg. Some of the retracted papers may have contained honest mistakes, some may have been negligent, still others were clearly purposeful. The latter is gravely unfair to honest readers as well as to honest reviewers. Medical journal editors must do more to protect readers from such bogus research. Scientific societies should urge their members to strictly comply with the International Committee of Medical Journal Editors (ICMJE) Recommendations for the Conduct, Reporting, Editing, and Publication of Scholarly Work in Medical Journals.^3^ Journals should make sure that only articles complying with those rules are accepted for publication. Medical societies in countries with particularly high rates of retracted articles should increase their efforts. Incentives which lead researchers toward fraudulence should definitely be removed.
